# The effects of compound stimulus extinction and inhibition of noradrenaline reuptake on the renewal of alcohol seeking

**DOI:** 10.1038/tp.2015.130

**Published:** 2015-09-01

**Authors:** T M Furlong, M J Pan, L H Corbit

**Affiliations:** 1 School of Psychology, University of Sydney, Sydney, NSW, Australia

## Abstract

Alcohol-related stimuli can trigger relapse of alcohol-seeking behaviors even after extended periods of abstinence. Extinction of such stimuli can reduce their impact on relapse; however, the expression of extinction can be disrupted when testing occurs outside the context where extinction learning took place, an effect termed renewal. Behavioral and pharmacological methods have recently been shown to augment extinction learning; yet, it is not known whether the improved expression of extinction following these treatments remains context-dependent. Here we examined whether two methods, compound–stimulus extinction and treatment with the noradrenaline reuptake inhibitor atomoxetine, would reduce the vulnerability of extinction to a change in context. Following alcohol self-administration, responding was extinguished in a distinct context. After initial extinction, further extinction was given to a target stimulus presented in compound with another alcohol-predictive stimulus intended to augment prediction error (Experiment 1) or after a systemic injection of atomoxetine (1.0 mg kg^−1^; Experiment 2). A stimulus extinguished as part of a compound elicited less responding than a stimulus receiving equal extinction alone regardless of whether animals were tested in the training or extinction context; however, reliable renewal was not observed in this paradigm. Importantly, atomoxetine enhanced extinction relative to controls even in the presence of a reliable renewal effect. Thus, extinction of alcohol-seeking behavior can be improved by extinguishing multiple alcohol-predictive stimuli or enhancing noradrenaline neurotransmission during extinction training. Importantly, both methods improve extinction even when the context is changed between extinction training and test, and thus could be utilized to enhance the outcome of extinction-based treatments for alcohol-use disorders.

## Introduction

The high incidence of relapse, even following long periods of abstinence, is a major obstacle for successful treatment of drug and alcohol abuse.^1^ Situational cues associated with drinking are powerful reminders of the effects of alcohol and can trigger craving and drug-seeking behaviors even after long periods of abstinence.^2, 3, 4, 5^ Alcohol-associated cues are, therefore, a major factor contributing to increased risk of relapse and reducing the impact of these cues should reduce relapse rates.

Behavioral extinction is one approach for reducing the influence of alcohol-related cues, in which repeated presentation of the cue without alcohol leads to a reduction in responses, such as craving, triggered by the cue. However, extinction-based treatments for addiction have had variable success and need to be improved.^6, 7, 8^

A potential explanation for the limited success of extinction-based therapies is that the expression of extinction is easily disrupted by re-exposure to the reward or associated stimuli, stress or simply the passage of time.^9^ These recovery phenomena demonstrate that extinction training does not erase the original learning, but rather produces new learning that competes to control expression of the original behavior, in this case alcohol seeking. Thus, extinction alone does not eliminate relapse risk. However, recent advances from the animal learning field indicate ways that behavioral extinction can be improved, which if applied to alcohol seeking, could better reduce relapse risk.

As extinction is a form of new learning, one approach for improving extinction is to utilize knowledge of the conditions that produce robust learning in general. Error-correction models of learning postulate that learning occurs when there is a discrepancy between what is expected, on the basis of the presence of predictors, such as conditioned cues, and what actually occurs (that is, the reward, in appetitive conditioning^10, 11, 12^). Within this framework, the presentation of multiple reward predictors followed by the absence of reward (extinction) would generate a larger prediction error than the presentation of a single predictor, alone, and consequently result in improved extinction learning. In line with this prediction, it has been shown that the simultaneous presentation of multiple cues during extinction training can enhance behavioral extinction relative to equivalent presentations of a single cue.^13, 14^

Another approach for improving extinction is the use of pharmacological agents that promote learning-related plasticity to regulate long-term learning and memory.^15, 16^ For example, drugs that increase activity of the neuromodulator noradrenaline, which is released in response to biologically significant, novel or surprising events and may engage arousal and attention mechanisms, can improve extinction.^14, 17, 18, 19, 20^

Although these findings have potential to improve the efficacy of extinction-based treatments, to improve the stability and longevity of extinction, another important feature of extinction that must be addressed is that the expression of extinction has often been shown to rely on the context in which it is tested.^9, 21, 22, 23, 24^ That is, changes in environmental stimuli from extinction training to test can result in a failure of extinction expression and a return of responding, an effect termed *renewal*. Contextual specificity may contribute to why current exposure therapies are not more successful and raises the possibility that, although treatments that deepen extinction learning may reduce relapse risk under some circumstances, if the expression of this extinction is still regulated by context, such treatments may have limited utility. That is, treatments that are effective within a clinical setting may not be equally effective when individuals return to their normal lives and encounter situational cues previously paired with alcohol use.

It remains to be determined whether deepened extinction, achieved through methods such as compound extinction or treatment with noradrenergic drugs, is restricted to the context where extinction learning took place. The aim of the following experiments was, therefore, to examine whether manipulations that have previously been shown to enhance extinction when training and testing occurs in the same context will also reduce recovery of responding, that is, renewal, when animals are tested outside of the extinction context. On the basis of previous findings, we predicted that when tested, animals would respond less to a stimulus that was extinguished in compound with an additional alcohol-predictive stimulus than to a stimulus that was only extinguished alone. Further, we predicted that treatment with the noradrenaline reuptake inhibitor atomoxetine during extinction training would reduce responding at test. The question of interest was whether the enhanced extinction produced by compound extinction or atomoxetine treatment is dependent on the context where extinction took place and is therefore subjected to renewal when the context is changed.

## Materials and methods

### Experiment 1: does deepened extinction produced by compound extinction survive a change in context?

#### Subjects

The subjects were 12 male Hooded Wistar rats purchased from Laboratory Animal Services at the University of Adelaide (Adelaide, SA, Australia), weighing ~450 g at the beginning of the experiment. They were housed in groups of three to four and were given unlimited access to laboratory chow and water in the home cage. They were kept in an air-conditioned colony room maintained on a 12-h light–dark cycle (lights on at 0700 hours). Experiments were carried out in the light cycle. All procedures were approved by the Animal Ethics Committee at the University of Sydney and were in accordance with the Guide for the Care and Use of Laboratory Animals.

#### Apparatus

Training and testing took place in 16 Med Associates (East Fairfield, VT, USA) operant chambers housed in sound- and light-resistant shells. Each chamber possessed a retractable lever that when pressed activated a pump fitted with a syringe that could deliver 0.1 ml of a 10% ethanol solution into a recessed magazine. The chambers also contained a white noise generator, a solenoid that, when activated, delivered a 5-Hz clicker stimulus, and round stimulus lights positioned on the same wall to either side of the magazine. Auditory stimuli were adjusted to 80 dB in the presence of a background noise of 60 dB provided by a ventilation fan. Microcomputers equipped with the MED-PC software (Med Associates) controlled all experimental events and recorded responses.

#### Contexts

Two distinct contexts were created by altering the visual, olfactory and tactile components of the chambers. Context A was constructed with laminated white sheets, with black-filled circles positioned on the transparent top, front and back walls of the chambers, 0.5 ml of 10% artificial rosewater essence on paper towel was placed beneath the floor (Queen Fine Foods, Queensland, NSW, Australia), which consisted of a series of steel rods. Context B was constructed with laminated sheets of black-and-white vertical stripes positioned on the walls, 0.5 ml of 10% vanilla essence beneath the floor and a smooth plastic tile floor. Acquisition trials took place in a context different from extinction trials and the specific identity of the context (A or B) was counterbalanced across rats.

#### Alcohol acclimation

To acclimate the rats to alcohol, 24-h access to a bottle of 10% ethanol was given in their home cages for 10 days. This was followed by 10 days of 1-h access to a bottle of 10% ethanol at the time when training would subsequently occur. Water was always available in a separate bottle. Rats were weighed daily and their ethanol and water consumption were determined by weighing the bottles to the nearest 0.1 g before and after each session.

#### Magazine and lever training

On the first training day, rats received a 30-min magazine training session during which 10% ethanol was delivered according to a random time 30-s schedule. On the next day, the lever was introduced and each press resulted in the delivery of 0.1 ml of 10% ethanol; however, there were no discriminative stimuli present. Animals that failed to acquire the instrumental response in this session were given addition days of training until a minimum of 20 alcohol deliveries were produced in one session. Magazines were inspected at the end of each session to ensure that all alcohol was consumed.

#### Acquisition

A summary of the design of Experiment 1 can be found in Table 1. The next day, discrimination training began. Each session consisted of 24 30-s presentations of either a light, white noise or clicker stimulus (eight trials each). During each stimulus, lever pressing resulted in 10% ethanol delivery. In order to aid acquisition, on the first day, the lever was inserted at the beginning and then retracted at the end of each stimulus. On subsequent days, the lever was present throughout the session and response during the stimuli was reinforced according to an increasing variable ratio schedule (days 1–3 fixed ratio (FR) 1; days 4–6 variable ratio (VR) 2; days 7–9 VR3), totaling nine reinforced training sessions with the three discriminative stimuli.

#### Extinction Phase 1

The following day, rats received 12 non-reinforced individual presentations of the noise and clicker and light cues (four per stimulus) in a random order. This was performed in the extinction context, which was distinct from the acquisition context as described above.

#### Extinction Phase 2

After Extinction 1, rats received six trials of each a stimulus compound (AX) and the remaining auditory stimulus (Y) in the extinction context.

*Tests.* One week later, rats were tested in both the acquisition and extinction contexts on separate days in a counterbalanced order. In each test, rats were placed into the context and lever-press responses were measured during three trials of each the noise and clicker (X and Y).

#### Sucrose reward

A separate group of 12 rats were trained to self-administer sucrose to verify the generality of any effects. Training and testing procedures were identical to those described above, except that animals were not exposed to alcohol and were trained to self-administer a 20% sucrose solution, and they underwent an additional session of extinction before the introduction of the compound stimulus to further reduce response rates, which were higher for sucrose than for ethanol.

### Experiment 2: does deepened extinction following atomoxetine treatment survive a change in context?

*Subjects and apparatus.* The subjects were 24 male Hooded Wistar rats weighing ~420 g at the beginning of the experiment. Rats were sourced and housed as described in Experiment 1. The apparatus and acclimation procedures were identical to those described in Experiment 1.

#### Magazine and lever-press acquisition

On the first training day, rats received a 30-min magazine training session, during which 10% ethanol was dispensed according to a random time 30-s schedule. The next day, rats underwent instrumental training. The levers were introduced and responses were reinforced with 10% ethanol according to a FR1 schedule. On subsequent days, response was reinforced according to an increasing variable ratio schedule (days 1–3 FR1; days 4–6 VR2; days 7–9 VR3). Sessions were 30 min in duration. No discrete stimuli were used in this experiment.

#### Extinction Phase 1

After acquisition, rats were placed in a distinct extinction context as described above, and received one 10-min session in which lever-press responding was recorded but not reinforced with ethanol.

#### Extinction Phase 2

The following day, 45 min before the training session rats were injected with atomoxetine (1.0 mg kg^−1^ intraperitoneal (i.p.), Tocris, Ellisville, MO, USA; dissolved in saline and administered in a volume of 1 ml kg^−1^) or an equivalent volume of saline. This dose was chosen on the basis of previous studies that found this dose to be effective in reducing spontaneous recovery of responding for food or cocaine.^14, 20^ Rats then underwent a 10-min extinction session in the extinction context.

*Tests.* One week later, rats underwent two tests across 2 days, one in the acquisition and one in the extinction contexts in a counterbalanced order. The tests were the same as the initial extinction sessions; the levers were introduced and response was recorded for 10 min.

#### Sucrose reward

A separate group of 24 rats were trained to self-administer sucrose again to verify the generality of any effects. Training and testing procedures were identical to those described above, except that animals were not exposed to alcohol and were trained to self-administer a 20% sucrose solution.

## Results

### Experiment 1: compound stimulus extinction enhances extinction and survives a change in context

#### Alcohol acclimation

During the first 10 days of alcohol acclimation, the animals' mean (±s.e.m.) intake of 10% ethanol over 24 h was 17.1 (1.6) ml. Over the next 10 days, their average intake over the 1-h period was 3.8 (0.4) ml, producing an average alcohol level of 0.76 (0.08) g kg^−1^ demonstrating that rats would voluntarily consume unsweetened alcohol.

#### Acquisition

**Figure 1:**
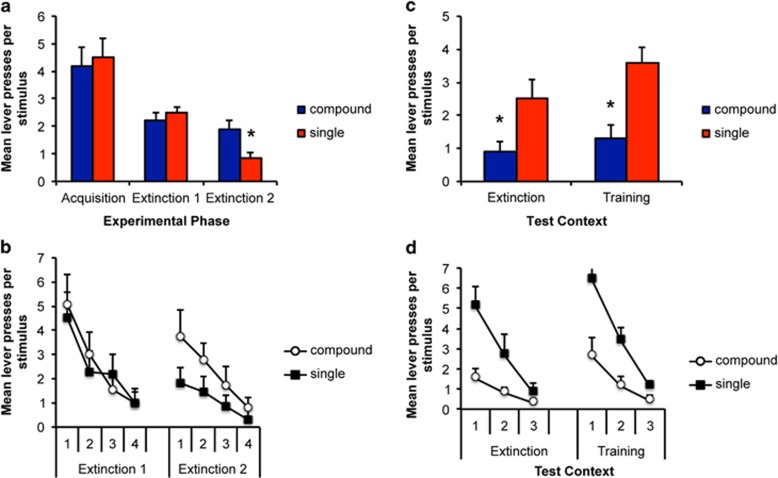
Extinction of a stimulus compound reduces recovery of alcohol seeking relative to extinction of a single stimulus in both the training and extinction contexts. (**a**) The mean lever presses per stimulus for the final acquisition and initial extinction sessions and in the session where the compound stimulus was introduced (Extinction 2). (**b**) Trial-by-trial data across extinction. (**c**) The mean lever presses per stimulus in the tests conducted 1 week later in either the training or extinction contexts. Presentation of a stimulus compound increased responding during extinction; however, when the element of that compound was tested alone 1 week later, animals responded less to the stimulus that was extinguished in compound than to the stimulus extinguished alone. There was no effect of the context in which rats were tested; thus, enhanced extinction produced by compound extinction is still expressed outside the extinction context. (**d**) Trial-by-trial data across tests in the extinction and training contexts. *Indicates a significant difference in number of lever presses; *P*<0.05; *N*=12.

Rats underwent 9 days of acquisition training, wherein a lever-press response in the presence of one of three discrete stimuli resulted in the delivery of alcohol. On the final day of training, the mean (±s.e.m.) lever presses per stimulus for the pre-stimulus, light, noise and clicker were 0.26 (0.08), 4.1 (0.5), 5.0 (0.1) and 4.2 (0.5), respectively. There were no differences between stimuli (F(2,11)<1). The data for the final day of training are illustrated in Figure 1a (Acquisition) as the mean response rates for the stimuli that would subsequently be presented as either single or compound during the final extinction session. There were no differences in response at this stage (F(1,11)=0.3, *P*>0.05).

#### Extinction

Rats subsequently underwent extinction in a distinct context. On the first day, there were 12 stimulus presentations and lever pressing was recorded, but no alcohol was delivered. Responding decreased from acquisition and there were no differences in the mean response rates for the stimuli that would subsequently be presented as either single or compound (Figure 1a, Extinction 1; F(1,11)=0.4, *P*>0.05). In the final extinction session, conditions were modified such that rats received six non-reinforced presentations of a compound stimulus (AX; light paired with one of the auditory stimuli, counterbalanced) interspersed with six individual stimulus (Y) presentations. As shown in Figure 1a (Extinction 2), rats responded significantly more to the stimulus compound than to the single stimulus (F(1,11)=6.9, *P*<0.05). The impact of extinction of the compound stimulus compared with continued extinction of a single stimulus on renewal of responding was assessed 1 week later. As shown in Figure 1c, at test, responding to the stimulus that had been extinguished as part of the compound (X) was lower than responding to the stimulus extinguished alone (Y) and this did not differ according to the context in which the test was conducted. This description is confirmed by the statistical analyses, which revealed a significant effect of stimulus (F(1,11)=15.9, *P*<0.01), but no effect of context (extinction versus training; F(1,11)=1.8, *P*>0.05) and no interaction between these factors (F(1,11)=1.4, *P*>0.05). These data provide further evidence that extinction of a stimulus compound can improve extinction of alcohol seeking and that this effect does not appear to be modulated by test context. However, there was little evidence of a context-based renewal effect in this paradigm.

### Sucrose reward

#### Acquisition

**Figure 2:**
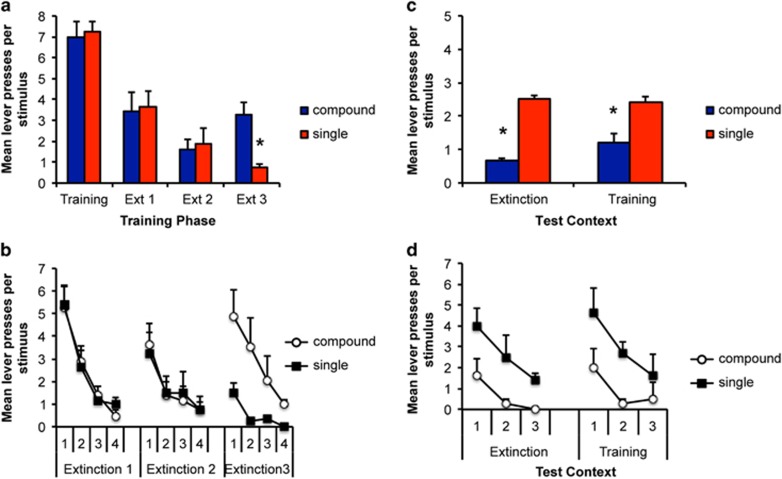
Extinction of a stimulus compound reduces recovery of sucrose seeking relative to extinction of a single stimulus in both the training and extinction contexts. (**a**) The mean lever presses per stimulus for the final acquisition and initial extinction sessions and in the session where the compound stimulus was introduced (Extinction 3). (**b**) Trial-by-trial data across extinction. (**c**) The mean lever presses per stimulus in the tests conducted 1 week later in either the training or extinction contexts. Presentation of a stimulus compound increased responding during extinction; however, when the element of that compound was tested alone 1 week later, animals responded less to the stimulus that was extinguished in compound than to the stimulus extinguished alone. There was no effect of the context in which rats were tested; thus, enhanced extinction produced by compound extinction is still expressed outside the extinction context. (**d**) Trial-by-trial data across tests in the extinction and training contexts. *Indicates a significant difference in number of lever presses; *P*<0.05; *N*=12.

On the final day of training, the mean (±s.e.m.) lever presses per stimulus were 0.7 (0.04), 6.0 (0.12), 7.4 (0.09) and 6.8 (0.12) for the pre-stimulus, light, noise and clicker, respectively. Importantly, there was no difference in responding to the stimulus that would subsequently be extinguished alone or as part of a compound at this stage (F(1,11)=0.3, *P*>0.05; Figure 2a).

#### Extinction

Figure 2a shows the animals' responding to the individual stimuli (X and Y) during the initial 2 days of extinction training (Extinction 1 and 2) and to the compound stimulus (AX) and to the single stimulus (Y) presented in isolation during the final extinction session (Extinction 3). During the initial extinction, there was a significant effect of day, indicating that responding decreased across extinction ((1,11)=11.9, *P*<0.01), but no significant difference in responding to the two stimuli, (F(1,11)=0.1, *P*>0.05) and no interaction between these factors (F(1,11)=0.3, *P*>0.05). In the third extinction session, extinguished responding was increased by compound stimulus presentation relative to the single stimulus for which responding remained low (F(1,11)=24.9, *P*<0.01). The data of primary interest are presented in Figure 2c, where rats were tested 1 week later in each of the extinction and training contexts to examine evidence of renewal. Rats showed greater recovery of responding to the stimulus that had been extinguished alone (Y) than in compound (X) (F(1,11)=8.1, *P*<0.05); however, there was no overall effect of context (F(1,11)=0.2, *P*>0.05) and no interaction with this factor (F(1,11)=0.7, *P*>0.05). Thus, as for alcohol reward, we show that extinction of sucrose seeking is enhanced by compound stimulus presentations and this effect does not appear to be modulated by context; however, again, there was little evidence of renewal in this paradigm.

### Experiment 2: atomoxetine enhances extinction and attenuates renewal

#### Alcohol acclimation

During the first 10 days of alcohol acclimation, the animals' average intake of 10% EtOH over 24 h was 10.3 (1.4) ml. Over the next 10 days, their average intake over the 1-h period was 3.6 (0.06) ml, producing an average alcohol level of 0.89 (0.03) g kg^−1^.

#### Acquisition

**Figure 3:**
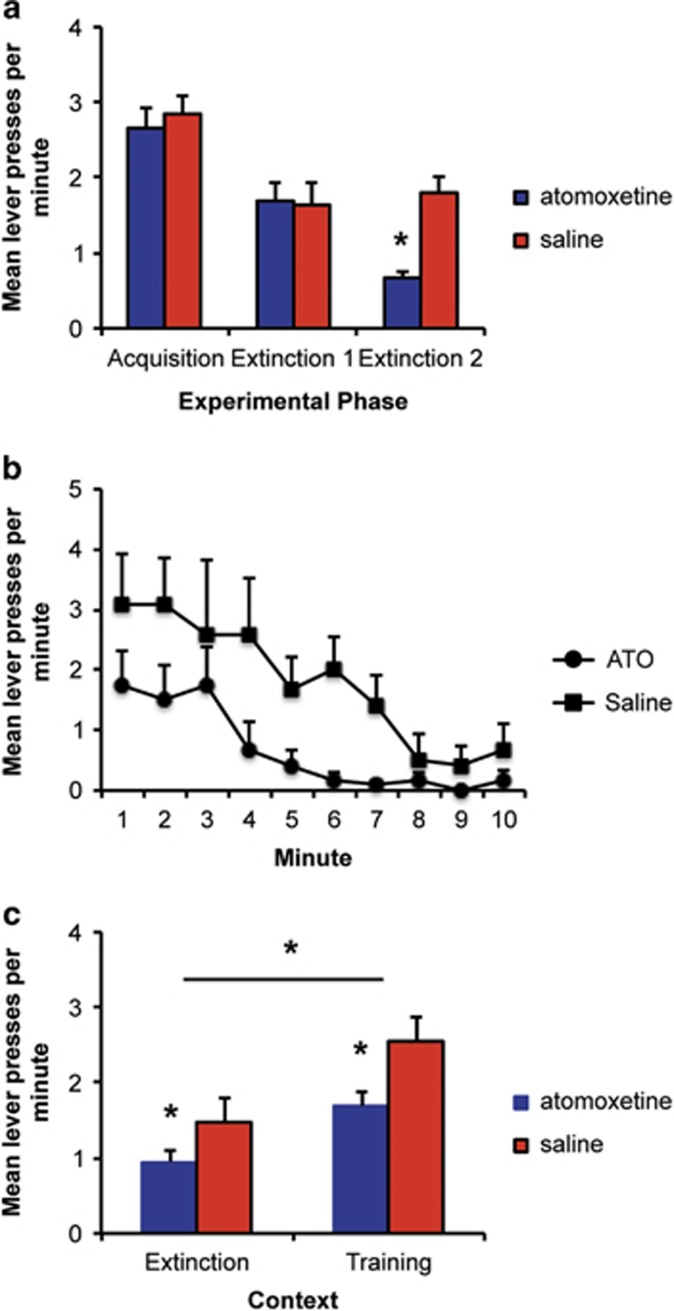
Atomoxetine treatment enhances the extinction of alcohol seeking when rats are tested in both the training and extinction contexts. (**a**) The mean lever presses per minute for the final acquisition and initial extinction sessions and in the final extinction session (Ext 2) when atomoxetine or saline was administered. There were no group differences during training or initial extinction. Atomoxetine reduced response during the final extinction session. (**b**) Minute-by-minute response during the final extinction session after administration of either atomoxetine or saline. Atomoxetine reduced responding during this session. (**c**) The mean lever presses per minute during the test sessions conducted drug free 1 week later in the training and extinction contexts. Rats responded more when returned to the training context; however, responding was lower for rats treated with atomoxetine in both the training and extinction contexts. Together, these results indicate that atomoxetine reduces recovery of alcohol seeking even when testing occurs in the drug-paired context. *Indicates a significant difference in number of lever presses; *P*<0.05; *N*=12 per group.

The mean lever presses in the final training session are shown in Figure 3a. There were no differences in response rates between animals subsequently assigned to receive atomoxetine versus saline (F<1). The mean number of alcohol deliveries across training were 31.9 (1.3), equivalent to 3.2 (0.13) ml and producing an average alcohol level of 0.64 (0.02) g kg^−1^.

#### Extinction

Rats decreased responding when alcohol was withheld and were assigned to groups matched for responding in this initial extinction session to ensure that there were no differences in responding between groups (Figure 3a, Extinction 1; F(1,22)<1). The next day, the rats were treated with either atomoxetine (1.0 mg kg^−1^, i.p.) or saline (1 ml kg^−1^, i.p.) 45 min before an additional extinction session where the lever was available for 10 min, but no alcohol was delivered (Figure 3a, Extinction 2). Responding was lower in rats treated with atomoxetine (F(1,22)=29.8, *P*<0.01). To further examine the nature of this effect, we examined lever pressing across minutes of the extinction session. As shown in Figure 3b, both the groups decreased responding across minutes of the test; however, responding was lower throughout for rats treated with atomoxetine. This was supported by an effect of minute (F(9,198)=4.9, *P*<0.01) and of group (F(1,22)=29.8, *P*<0.01); however, there was no interaction between these factors (F<1).

#### Test

Animals were tested in both the training and extinction contexts 1 week later in a counterbalanced order. As shown in Figure 3c, rats responded more in the training context than in the extinction context, thus showing evidence of renewal (F(1,22) 12.7, *P*<0.01). There was also a main effect of group indicating that rats that received atomoxetine during extinction training responded less at test (F(1,22)=5.5, *P*<0.05). There was no interaction between these factors (F(1,22)=0.3, *P*>0.05) indicating that, although animals still responded more in the training context, thus showing evidence of renewal, atomoxetine was effective in reducing responding in both the extinction and training contexts. Importantly, the finding that animals treated with atomoxetine responded less in the training context than those treated with saline suggests that the ability of atomoxetine to improve extinction is not eliminated by a context shift.

### Sucrose reward

#### Acquisition

On the final day of training, responses per minute were 8.2 (0.33) and 8.3 (0.28) and rats earned 27 and 31 reinforcers for the atomoxetine and saline groups, respectively. There were no group differences in acquisition (Fs<1).

#### Extinction

**Figure 4:**
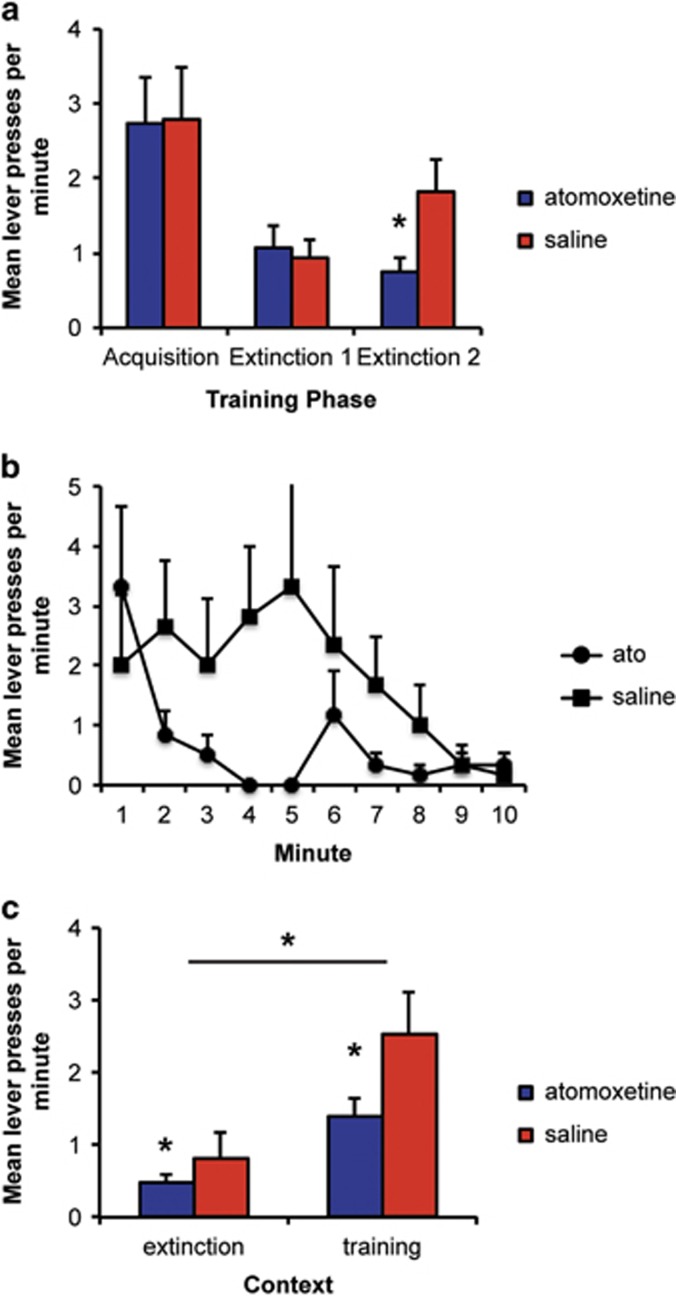
Atomoxetine treatment enhances extinction of sucrose seeking when rats are tested in both the training and extinction contexts. (**a**) The mean lever presses per minute for the final acquisition and initial extinction sessions and in the final extinction session (Ext 2) when atomoxetine or saline was administered. There were no group differences during training or initial extinction. Atomoxetine reduced responding during the final extinction session. (**b**) Minute-by-minute responding during the final extinction session after administration of either atomoxetine or saline. Atomoxetine reduced responding during this session. (**c**) The mean lever presses per minute during the test sessions conducted drug free 1 week later in the training and extinction contexts. Rats responded more when returned to the training context; however, responding was lower for rats treated with atomoxetine in both the training and extinction contexts. Together, these results indicate that atomoxetine reduces recovery of sucrose seeking even when testing occurs in the drug-paired context. *Indicates a significant difference in number of lever presses; *P*<0.05; *N*=12 per group.

Rats decreased response when the sucrose solution was no longer delivered and were assigned to groups based on their performance in this session (Figure 4a, Extinction 1; F(1,22)<1). The next day, rats were treated with either atomoxetine (1.0 mg kg^−1^, i.p.) or saline (1 ml kg^−1^, i.p.) as in the ethanol experiment. Responding decreased across minutes (Figure 4b; F(1,22)=3.4, *P*<0.05), and whereas both groups decreased responding across minutes, overall, responding was lower in rats treated with atomoxetine. This was supported by an effect of group (F(1,22)=4.9, *P*<0.05); however, the interaction between group and minute was not significant (F(1,22)=1.7, *P*<0.05).

#### Test

As shown in Figure 4c, rats responded more in the training context than in the extinction context, thus showing evidence of renewal (F(1,22) 29.1, *P*<0.01). There was also a main effect of group indicating that rats that received atomoxetine during extinction training responded less at test overall (F(1,22)=8.1, *P*<0.01). There was no interaction between these factors (F(1,22)=2.8, *P*>0.05), indicating that, despite evidence of renewal, atomoxetine was effective in reducing responding in both the extinction and training contexts.

## Discussion

In abstinent alcoholics, exposure to alcohol cues increases alcohol craving and physiological and negative affective responses, which are associated with vulnerability to alcohol relapse.^4^ Decreasing the conditioned properties of these cues, therefore, may reduce relapse risk. However, cue-exposure treatments that aim to reduce responses to such cues have found limited success.^7^ An important factor that may contribute to the limited efficacy of cue-exposure treatments is that the expression of extinction may rely on the context in which it is tested. In relation to addiction, people use alcohol and drugs in the presence of a range of environmental and social stimuli; however, extinction training, presumably as part of treatment, occurs in a distinct laboratory or clinic setting. Thus, it is possible that extinction is disrupted outside that setting when people return to their daily lives and re-encounter previously drug-predictive cues. Whereas both behavioral and pharmacological treatments have been shown to deepen extinction of food- and drug-seeking behaviors and thus indicate ways that current extinction-based therapies could be improved,^13, 20^ to date there is little research to address whether these treatments will remain effective when testing occurs outside the environment where extinction took place. Such evidence is critical for clinical application as treatments that are not resilient enough to withstand changes in context, so as to generalize outside the clinic setting, will be of limited utility. The aim of the current study was, therefore, to determine whether deepened extinction effects are context-dependent.

In Experiment 1, rats learned to make a lever-press response to earn alcohol in the presence of three discrete stimuli. Responding to the stimuli was then extinguished in a distinct context and, after initial extinction, rats underwent additional extinction trials where two stimuli were presented together, in compound, and the remaining stimulus continued to be extinguished alone. At test, responding was lower to the stimulus that had been extinguished as part of a compound, providing evidence that the combined extinction of multiple alcohol-predictive stimuli improves extinction of those stimuli relative to the same amount of extinction of a single stimulus alone. Importantly, for the first time, we demonstrate that this effect is not compromised when testing occurs outside the context where extinction learning took place; a stimulus extinguished as part of a compound produced less responding than one extinguished only alone whether tested in the training or extinction context. However, whereas returning the animals to the training context for testing did not eliminate the benefit of compound extinction, a potential limitation of these findings is that there was little evidence of renewal using these procedures, and so whether deepened extinction would still be evident in the face of stronger renewal was unclear.

There are several likely reasons for the lack of renewal in this paradigm. First, the use of a discriminative stimulus procedure meant that response was only reinforced in the presence of discrete stimuli, which may have been more salient than the background context. Thus, the discrete stimuli may have overshadowed the contextual stimuli with the result that contextual information had less predictive value and had little role in modulating responding. Second, acquiring the task involved learning that the response was only reinforced during the stimuli and not in the context alone, and so animals in fact had significant experience with extinction of the response in the training context (if they responded in the absence of the discrete stimulus). This may have established an inhibitory association between the training context and the response and eliminated renewal.^25^ Finally, renewal effects typically do not restore responding to training levels, indicating that only a portion of extinction learning is context-dependent^9^ and these methods may have produced learning that is largely context-independent.

Experiment 2 used a different design to examine whether enhanced extinction effects could be found in a paradigm that produced more robust renewal. Specifically, rats were trained to make a lever-press response for alcohol reward in a distinct context in the absence of any discrete stimuli. Responding was subsequently extinguished in a different context and then tested in both the training and extinction contexts. As no discrete stimuli were used because of the lack of renewal in Experiment 1, we instead turned to a different method previously shown to improve extinction—treatment with the noradrenaline reuptake inhibitor, atomoxetine. Our findings replicate previous demonstrations that rats treated with atomoxetine respond less when tested in same context where they were extinguished compared with saline controls.^14^ Importantly, we found that, although overall rats responded more in the training context, atomoxetine reduced alcohol seeking relative to controls indicating improved extinction despite the shift in context.

In terms of clinical application, it may seem problematic that responding increased when rats were returned to the training context for testing, even in the atomoxetine group. However, parameters were chosen in part to avoid floor effects and ensure that a renewal effect would be seen in the control group to allow comparison with atomoxetine treatment, and to address our primary question of whether any benefit from these treatments would be context-dependent. The current data provide evidence that these effects are resistant to context change. Future studies can investigate whether parameters (amount of extinction, repeated treatment with atomoxetine) can be optimized to eliminate recovery of responding entirely, which would have greater clinical utility. Alcohol-associated cues contribute to increased risk of relapse, and increased limbic activation to alcohol cues predicts relapse in recovering alcoholics.^26, 27^ For example, in abstinent alcoholics, striatal and other limbic activation not only correlates with cue-induced craving; importantly, this activity is reduced by successful treatments including cue-exposure therapies.^28, 29^ These findings suggest that inhibition of cue-related activity is critical for abstinence. The prefrontal cortex in particular may be important for controlling drug use^30^ and other work indicates structural abnormalities in the prefrontal cortex of alcoholics,^31, 32^ which may relate to impairments in adapting their behavior to changing reward contingencies and controlling the influence of cravings.^32, 33^ In a recent functional magnetic resonance imaging study, atomoxetine was shown to accelerate extinction of cue–outcome associations and this effect was accompanied by increased activation of the hippocampus and ventromedial prefrontal cortex, regions associated with extinction learning,^34, 35^ as well as insula and anterior cingulate cortices, regions implicated in error detection and processing of changing reinforcement contingencies.^36^ Other studies have shown deficits in working memory and attentional tasks in abstinent alcoholics,^37^ consistent with impaired executive function and dysfunctional of prefrontal cortical control in addiction.^30^ Furthermore, ability to activate prefrontal cortical areas under high working memory load predicted future abstinence in detoxified alcohol-dependent patients.^38^ Thus, treatment with drugs such as atomoxetine may have broad effects on behavior and help ameliorate cognitive deficits particularly in early abstinence by improving impaired cortical function, and given the established importance of noradrenaline in attention.^39^

Of interest for the translation of the current findings to clinical populations, atomoxetine has been shown to reduce the cumulative heavy-drinking days in a population with comorbid attention-deficit/hyperactivity disorder and alcohol-use disorders.^40^ Although this treatment did not reduce the time to relapse, atomoxetine was given without any other psychosocial treatment and, therefore, it is possible that atomoxetine in conjunction with exposure therapy or other cognitive behavioral intervention may improve attention and engagement with treatment to further improve treatment outcomes. Furthermore, the lack of adverse events in this study establishes that atomoxetine can be used safely within this patient population even under conditions where patients are at risk for relapse to alcohol use.^40^ Thus, advances in behavioral procedures and the addition of pharmacological adjuncts within the clinic setting may be able to safely augment the extinction learning that occurs as part of treatment to ultimately improve treatment outcomes, and the current data suggest that these effects should generalize outside the clinic setting. Trials examining the effects of atomoxetine in a population with alcohol-use disorders but without comorbid attention-deficit/hyperactivity disorder will be an important first step in this regard.

Extinction-based treatments in clinical samples have had variable success,^8^ however, have shown more promising effects in alcoholics than other drug users and are more effective when used in a sample identified as cue-reactive before intervention.^41^ It is more likely that the benefits to extinction identified here will extend to patients identified as cue-reactive than to those that are not. Furthermore, it is acknowledged that alcoholism itself is a heterogeneous disorder and that no single treatment is likely to be effective for all.^42^ Nonetheless, identifying new treatment approaches, and particularly the combination of behavioral and pharmacological interventions, may be of considerable benefit for some individuals suffering from alcohol-use disorders. Together, our findings indicate that compound stimulus presentation or increased noradrenergic activity during extinction learning reduced the subsequent return of drug seeking, and importantly, demonstrate that these effects were still evident when animals were tested outside the extinction context. This has important implications for clinical application of such methods as it addresses the concern that extinction therapies may have limited utility outside the clinic setting and suggests that these are promising methods for improving the efficacy of extinction treatments.

## Figures and Tables

**Table 1:** Design of Experiment 1

*Acquisition*	*Extinction 1*	*Extinction 2*	*Delay (days)*	*Test*
A+	A−	AX−	7	X−
X+	X−	Y−		Y−
Y+	Y−			

A represents the light stimulus, and X and Y represent the noise and clicker stimuli, respectively (counterbalanced). '+' indicates that responding during the stimuli resulted in delivery of 0.1 ml of 10% alcohol (or 20% sucrose) as the reinforcer. '−' indicates that reinforcer was not delivered.
